# Art therapy as an adjuvant treatment for schizophrenia: A protocol for an updated systematic review and subgroup meta-analysis of randomized clinical trials following the PRISMA guidelines

**DOI:** 10.1097/MD.0000000000030935

**Published:** 2022-10-07

**Authors:** Xuexing Luo, Zheyu Zhang, Zhong Zheng, Qian Ye, Jue Wang, Qibiao Wu, Guanghui Huang

**Affiliations:** a Faculty of Humanities and Arts, Macau University of Science and Technology, Taipa, Macau, China; b College of Art and Design, Wuhan Technology and Business University, Wuhan, Hubei, China; c Jingdezhen China Ceramics Museum, Jingdezhen, Jiangxi, China; d State Key Laboratory of Quality Research in Chinese Medicines, Macau University of Science and Technology, Taipa, Macau, China; e Faculty of Chinese Medicine, Macau University of Science and Technology, Taipa, Macau, China; f Guangdong-Hong Kong-Macao Joint Laboratory for Contaminants Exposure and Health, Guangzhou, Guangdong, China.

**Keywords:** art therapy, complementary therapy, meta-analysis, schizophrenia, systematic review

## Abstract

The efficacy of art therapy as an adjuvant treatment for schizophrenia remains inconclusive, and variation in the effects of art therapy on schizophrenia by the type of schizophrenia, severity of schizophrenia, type of art therapy, follow-up duration, or different populations has never been systematically assessed. The objective of this study is to systematically evaluate the effects of art therapy on schizophrenia and to determine whether there are some potential influencing factors affecting the effects of art therapy.

**Methods:**

Seven online databases will be searched from their inception until June 30, 2022. All the relevant randomized clinical trials (RCTs) comparing art therapy plus standardized treatment versus standardized treatment alone for schizophrenia will be selected and assessed for inclusion. The Cochrane risk-of-bias tool will be used to evaluate the methodological quality of the included RCTs. Review Manager 5.4 will be used to analyze all the data obtained. Mental health symptoms are defined as the primary outcome, and the secondary outcomes include the Global Assessment of Functioning score, quality of life, functional remission, and the level of self-esteem. Subgroup analyses will be performed based on the type of schizophrenia, severity of schizophrenia, type of art therapy, follow-up duration, or different populations.

**Results:**

The results will be published in a peer-reviewed journal.

**Conclusions:**

This updated systematic review and subgroup meta-analysis will evaluate the effects of art therapy as adjunctive treatment to standardized treatment in patients with schizophrenia and determine whether there are some potential confounding variables affecting the effects of art therapy on the outcomes of schizophrenia patients, thus strengthening the evidence base for the clinical application of this combination therapy for schizophrenia.

## 1. Introduction

Schizophrenia is a chronic and severely disabling mental disorder affecting approximately 1 in 300 people (0.32%) or 24 million people worldwide^[1–3]^ and is characterized by significant impairments in the perception of reality and changes in behavior. The common symptoms of schizophrenia are associated with emotional, cognitive, and psychosocial dysfunctions, such as delusions, hallucinations, disorganized thoughts and speech, disorganized behavior, and negative symptoms (decreased emotional affect or response, lethargy, etc).^[1–3]^

With the advent of antipsychotic drugs and nondrug treatments, the prognosis of the disease has improved, but long-term medication is prone to lead to reduced compliance, adverse reactions, and impairment of social function.^[4–6]^ Standardized treatment for schizophrenia refers to drug therapy combined with nondrug therapy, such as electroconvulsive and transcranial magnetic stimulation, supplemented by psychosocial therapy, including behavioral therapy (social skills training), family intervention, community service, etc.^[7–10]^ At present, it is believed that adjuvant psychotherapy can improve social function, among which art therapy has attracted the attention of scholars in this field. The British Association of Art Therapists defines art therapy as a process in which patients express their unexpressed thoughts and emotions through works of art and interact with therapists.^[11]^ Art therapy has been increasingly used as an adjuvant treatment for schizophrenia patients since it was first reported by Adrian Hill in 1942. The types of art therapies include music therapy, dance therapy, color therapy, clay therapy, drama therapy, play therapy, Mandala therapy, etc.^[12–15]^

Art therapy has been increasingly recognized as a practical and effective treatment for diverse types of mental and physical conditions.^[16]^ For example, art therapy has been shown to bring benefits to patients with schizophrenic disorders, traumatic brain injury, mammary cancer, posttraumatic stress disorder,^[17,18]^ and numerous other conditions. However, the evidence for the use of art therapies for schizophrenia is still extremely weak; only a meta-analysis reported that art therapy had only a “small” therapeutic effect for negative symptoms, which was limited to lower quality trials or trials without blinding assessment of outcomes.^[19]^ Whether art therapy can improve other outcomes of schizophrenia remains unclear. In addition, this meta-analysis only searched PubMed and Scopus for studies published in English and included only 9 randomized clinical trials (RCTs). RCTs published in other databases or languages were not included. In fact, there are still dozens of other eligible RCTs that met the inclusion criteria and should be included in the meta-analysis.^[20–24]^ The selective and incomplete inclusion of RCTs might have undermined the strength of the evidence of this study. Furthermore, whether the type of schizophrenia, severity of schizophrenia, type of art therapy, follow-up duration, or different subgroups of patients are associated with the effectiveness remains unknown, and subgroup analyses based on these variables may help address the issue.

This updated systematic review and subgroup meta-analysis will include more eligible RCTs to systematically evaluate the effects of art therapy on more outcomes of a larger population with schizophrenia and determine the possible variation in the effects of art therapy on schizophrenia by the type of schizophrenia, severity of schizophrenia, type of art therapy, follow-up duration, or study population, aiming to provide stronger evidence for the application of art therapy as an adjuvant treatment for schizophrenia.

## 2. Materials and Methods

### 2.1. Study registration

This study was registered as PROSPERO (International prospective register of systematic reviews) on July 17, 2022, registration number CRD42022344693 (https://www.crd.york.ac.uk/prospero/display_record.php?RecordID=344693). We will perform this updated systematic review and subgroup meta-analysis following the PRISMA (Preferred Reported Items for Systematic Review and Meta-analysis) guidelines.^[25]^

### 2.2. Types of studies

All randomized controlled trials (RCTs) that compared art therapy combined with standardized treatment versus standardized treatment alone will be selected and evaluated for potential inclusion in this research.

### 2.3. Types of participants

#### 2.3.1. Inclusion criteria.

The subjects included in the present research must meet the following criteria: a formal diagnosis of schizophrenia, no restrictions on gender, age, ethnicity and disease course, the diagnosis of schizophrenia conforms to the Chinese Classification and Diagnostic Criteria for Mental Disorders 3rd edition (CCMD-3) or CCMD-2 or ICD-10,^[26]^ the 5th edition of the American Diagnostic and Statistical Manual of Mental (DSM-V),^[27]^ or other diagnostic criteria; art therapy adjunctive interventions must comply with the British Association of Art Therapists definition; an intervention of art therapy, both individual or group, which could also include variations, for example, color therapy, dance therapy, music therapy, play therapy; reporting means, standard deviation and sample size for a measure of negative symptoms, for example, Positive and negative syndrome scale,^[28]^ Global assessment of functioning score (GAF),^[29,30]^ Self-esteem scale (SES),^[31]^ and the functional remission of general schizophrenia (FROGS).^[32]^

#### 2.3.2. Exclusion criteria.

The clinical studies were not RCTs; the diagnosis was not schizophrenia; trials had inconsistent participant baseline data; and there were no measurements of related outcomes, unextractable data or unavailable full text.

### 2.4. Types of interventions

Art therapy combined with standardized treatment was used for the experimental group, while the control group was treated with standardized treatment alone.

### 2.5. Types of outcome measures

Positive and negative psychotic symptoms and GAF (measured using the GAF Scale) are defined as the primary outcomes, and the secondary outcomes include quality of life (QOL), functional remission, and the level of self-esteem.

### 2.6. Information source

Two independent reviewers (XL and JW) will carry out a comprehensive literature search to retrieve and screen all the studies published in common databases, including PubMed, Scopus, Web of Science, Cochrane Library, Excerpt Medica Database (Embase), ClinicalTrials.gov, Wanfang Databases, and China National Knowledge Infrastructure (CNKI), from their inception to June 30, 2022. Our search strategies used for English databases or Chinese databases are shown in Table 1.

**Table 1:** Search strategies for English databases or Chinese databases.

Number	Search terms
#1	Art Therapy [MeSH]
#2	Color Therapy [MeSH]
#3	Dance Therapy [MeSH]
#4	Music Therapy [MeSH]
#5	Play Therapy [MeSH]
#6	#1 OR #2 OR #3 OR #4 OR #5
#7	Schizophrenia, schizophrenias [MeSH]
#8	Schizophrenic Disorders [MeSH]
#9	Schizophrenic Disorder [MeSH]
#10	Disorders, Schizophrenic [MeSH]
#11	Disorder, Schizophrenic [MeSH]
#12	#7 OR #8 OR #9 OR #10 OR #11
#13	#6 AND #12
#14	yishu zhiliao (art therapy)
#15	yanse liaofa (color therapy)
#16	wudao liaofa (dance therapy)
#17	yinyue liaofa (music therapy)
#18	youxi liaofa (play therapy)
#19	#14 OR #15 OR #16 OR #17 OR #18
#20	jingshenfenliezheng (schizophrenia)
#21	xijueshitiaozheng (schizophrenia)
#22	#20 OR #21
#23	#19 AND #23

### 2.7. Study selection

**Figure 1.**
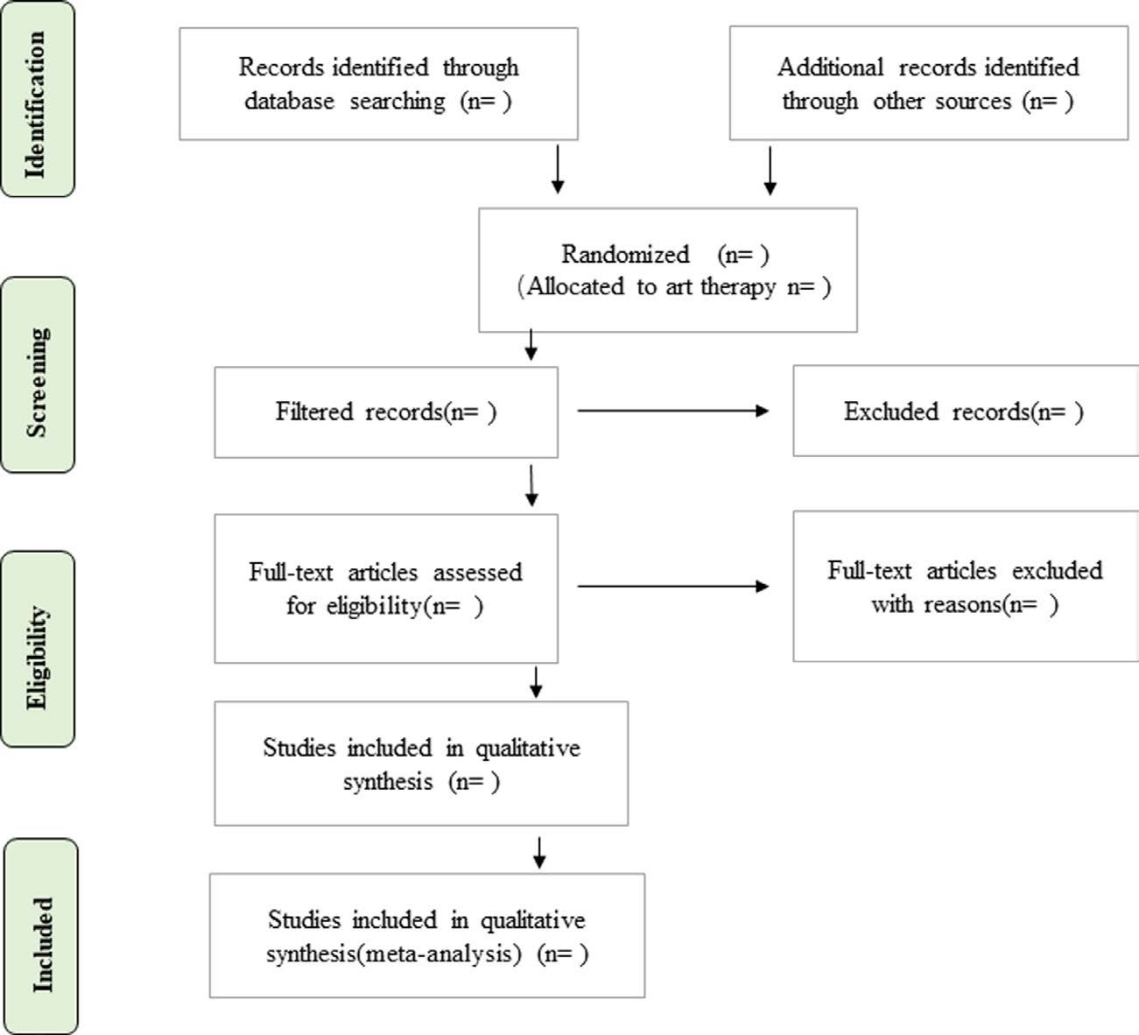
Flow diagram of the study selection process.

Based on the titles and abstracts, 2 independent investigators (QW and GH) will screen all the candidate articles. Then, the full texts will be retrieved for further assessment according to the inclusion and exclusion criteria. All inclusion debates will be resolved by discussion. The flow diagram of this study selection (Fig. 1) shows the data screening process of this study.

### 2.8. Data extraction

As mentioned above, 2 independent investigators will screen all the candidate articles on the basis of titles and abstracts and retrieve the full texts for further evaluation according to the inclusion/exclusion criteria. Disagreements on inclusion will be resolved by consensus. Three investigators (XL, QW, and GH) will independently assess the quality of the included studies and finish data extraction.

### 2.9. Assessment of risk of bias

Two independent reviewers (XL and JW) will use the Cochrane Risk of Bias Tool for Randomized Controlled Trials to determine the risk of bias in the included trials.^[33]^ The criteria used to evaluate the risk of bias in each trial are as follows: generation of random sequence; allocation concealment; blinding of personnel and participants; blinding of outcome evaluation; selective reporting; incomplete data; and other biases. The risk of bias will be graded as “high”, “unclear”, or “low”.^[34]^

### 2.10. Patient and public involvement

Patients and/or the public were not involved in the design, or conduct, or reporting, or dissemination plans of this research.

### 2.11. Strategy for data synthesis

A quantitative synthesis will be used if the included studies are sufficiently homogenous. Review Manager (RM) 5.3 (Copenhagen: The Nordic Cochrane Centre, The Cochrane Collaboration, 2014), Trial Sequential Analysis (TSA) software (Copenhagen Trial Unit, Center for Clinical Intervention Research, Copenhagen, Denmark; 2011), and Comprehensive Meta-Analysis (CMA) 3.0 (Biostat, Englewood, NJ; 2016) will be used to perform the analyses. Continuous data will be shown as weighted mean differences (WMDs) or standardized mean differences (SMDs),^[33,35,36]^ and dichotomous data will be shown as risk ratios (RRs), odds ratios (ORs), or risk differences (RDs) with their 95% confidence intervals (CIs).^[37–39]^ Statistical heterogeneity will be assessed using the *I*² statistic and *χ*² test, and when *I*^2 ^> 50% or *P* < .05, there is substantial heterogeneity, and a random-effects model will be applied; otherwise, a fixed-effects model will be applied to combine the data.^[40,41]^

### 2.12. Risk of bias across trials

When the number of RCTs outreaches or equals 10, Egger’s test and funnel plots will be applied to examine the potential bias in the RCTs included in the meta-analysis.^[33,42]^

### 2.13. Additional analyses

The required information size and the robustness of the meta-analysis results will be examined using TSA software, subgroup analysis, and sensitivity analysis.^[43]^ Subgroup analyses will be performed based on the type of schizophrenia, severity of schizophrenia, type of art therapy, follow-up duration, or study population.

### 2.14. Quality of evidence

The grading of recommendations, assessment, development and evaluations (GRADE) system will be used to evaluate the quality of evidence for each outcome by 2 independent reviewers (XL and JW).^[44]^

## 3. Results

The results will be published in a peer-reviewed scientific journal.

## 4. Discussion

Schizophrenia is a chronic and recurrent psychosis that has no cure, even with a good response to initial standardized treatment. Most schizophrenia patients have multiple relapses over time and experience intermittent or worsening symptoms. The medications for schizophrenia antipsychotic drugs are a must for the treatment of schizophrenia, but an increasing number of studies show that patients can also benefit from other types of therapy, including art therapy.

Art therapy, as one of the nonpharmacological complementary therapies, has been clinically used as an adjuvant treatment added to the standardized treatment for schizophrenia. However, evidence of this combination therapy is still lacking. This updated systematic review and meta-analysis will combine more related RCTs and participants to assess the effects of art therapy on schizophrenia patients. Furthermore, this is the first comprehensive subgroup meta-analysis of art therapy for schizophrenia based on the type of schizophrenia, severity of schizophrenia, type of art therapy, follow-up duration, and patient characteristics.

## 5. Conclusions

This updated systematic review and subgroup meta-analysis will evaluate the effects of art therapy as adjunctive treatment to standardized treatment in patients with schizophrenia and determine the possible variation in the effects of art therapy on schizophrenia, thus providing valuable evidence and guidance for the clinical application of this combination therapy for schizophrenia.

## Author contributions

**Conceptualization:** Qibiao Wu, Jue Wang, Guanghui Huang.

**Data curation:** Qibiao Wu, Xuexing Luo, Zheyu Zhang, Jue Wang.

**Formal analysis:** Qibiao Wu, Xuexing Luo, Jue Wang.

**Funding acquisition:** Qibiao Wu.

**Investigation:** Qibiao Wu, Xuexing Luo, Zheyu Zhang, Zheng Zhong, Ye Qian, Jue Wang, Guanghui Huang.

**Methodology:** Qibiao Wu, Xuexing Luo, Jue Wang.

**Project administration:** Qibiao Wu, Guanghui Huang.

**Resources:** Qibiao Wu, Guanghui Huang.

**Software:** Qibiao Wu, Xuexing Luo, Zheyu Zhang, Jue Wang.

**Validation:** Qibiao Wu, Xuexing Luo, Zheyu Zhang, Jue Wang, Guanghui Huang.

**Visualization:** Qibiao Wu.

**Writing – original draft:** Xuexing Luo, Zheyu Zhang.

**Writing & editing:** Qibiao Wu, Jue Wang, Guanghui Huang.
